# Microfluidic Viscometer Using a Suspending Micromembrane for Measurement of Biosamples

**DOI:** 10.3390/mi11100934

**Published:** 2020-10-14

**Authors:** Lelin Liu, Dinglong Hu, Raymond H. W. Lam

**Affiliations:** 1 Department of Biomedical Engineering, City University of Hong Kong, Hong Kong, China; raphael.liu@my.cityu.edu.hk (L.L.); hudinglong@genomics.cn (D.H.); 2 Institute of Biointelligence Technology, BGI-Shenzhen, Shenzhen 518083, China; 3 City University of Hong Kong Shenzhen Research Institute, Shenzhen 518057, China; 4 Centre for Biosystems, Neuroscience, and Nanotechnology, City University of Hong Kong, Hong Kong, China; 5 Centre for Robotics and Automation, City University of Hong Kong, Hong Kong, China

**Keywords:** microfluidic, viscosity, sensor

## Abstract

The viscosity of biofluids such as blood and saliva can reflect an individual’s health conditions, and viscosity measurements are therefore considered in health monitoring and disease diagnosis. However, conventional viscometers can only handle a larger liquid volume beyond the quantity that can be extracted from a person. Though very effective, micro-sensors based on electrokinetic, ultrasonic, or other principles often have strict requirements for the supporting equipment and complicated procedures and signal processing. Sample contamination is always an important issue. In this paper, we report a microfluidic viscometer requiring a small volume of biosamples (<50 µL) and straightforward operation procedures. It is fabricated with low-cost and biocompatible polymeric materials as one-time-use devices, such that contamination is no longer the concern. It contains a suspending micromembrane located along a microchannel. Under a steady driving pressure, the membrane displacement is a function of viscosity of the liquid sample being tested. We derived a simple analytical relation and perform a simulation for converting the membrane displacement to the sample viscosity. We conducted experiments with liquids (water and mineral oil) with defined properties to verify such a relation. We further applied the micro-viscometer to measure bovine blood samples with different hematocrit levels. It can be concluded that the microfluidic viscometer has a high compatibility with a broad range of biomedical applications.

## 1. Introduction

Viscosity is an essential fluid property. Besides reflecting the atomic and molecular composition of fluids, viscosity is of vital importance as a physical parameter in early biomedical diagnoses to examine the effect of physiological and pathological states of extracellular fluids on their chemical solubility [1], specific volume of floating cells [2], etc. For example, the blood viscosity can suggest the density of red blood cells and the underlying oxygen-carrying capacity [3]. The viscosity of gastric mucin reduces upon exposure to oxygen radicals due to the resultant weakened lipid binding capability [4]. The rheology of saliva can be affected by multiple factors such as calculus formation, caries, and even enamel solubility [5].

Traditional viscosity measurement schemes include mainly the rotational and capillary viscometers [6]. The rotational viscometer senses the torque of a rotating spindle immersed in the liquid being tested [7]. The capillary viscometer determines the liquid viscosity by measuring the time required for the liquid with a defined volume to flow through a capillary tube under a steady pressure [8]. Although these methods provide very reliable measurements, they both require a sample volume over milliliters, which is significantly beyond the typical amount of bioliquids that can be extracted from patients in clinical diagnostic applications. Considering that the volume of bio-samples (e.g., blood, saliva, sweat) obtained from patients is limited, micro-viscometers need to be developed to handle biosamples with a sub-milliliter volume for biomedical applications [9].

In recent years, there have been micro-viscometers developed based on various working principles. Electrokinetic or electrochemical micro-sensors have been developed for viscosity measurement [10]. Typically, multiple electrodes along the flow stream measure the potential differences between different upstream and downstream locations in order to estimate the liquid viscosity [11]. While the electrokinetic flow sensors offer measurement accuracy and linearity, the directly applied electrical signals can cause molecular damages, chemical alterations, or unexpected chemical reactions. Heating of liquids is another technical issue, especially for conductive samples (e.g., solvents with high ion concentrations). There are also micro-devices developed based on other detection schemes. For example, Yusop et al. developed a fluorescein whose fluorescent intensities vary with viscosity, yet photo-bleaching can be an important concern [12]. Yu et al. developed a micromachined capacitive ultrasonic transducer that obtains viscosity information by pulse-echo ultrasound signals under a damping effect [13]. One concern is that the ultrasound may trigger cell responses in the cell-containing biosamples.

Micro-structured mechanical sensors often appear to induce minimal effects in biosamples, in which any physical or biochemical stimuli can alter the sample conditions. Fedorchenko et al. reported a resonation-based micro/nano-cantilever with its maximum vibrational amplitude, bandwidth, and resonance frequency shift dependent on the liquid viscosity [14]. Kuoni et al. developed another resonation-based micro-viscometer using piezoelectric thin films fabricated on silicon membranes [15]. Although such measurement schemes are very effective, their corresponding complicated operation and signal processing may also be a hurdle toward practical bio-liquid measurement. Alternatively, passive micro-structured viscosity sensors can offer simpler operation procedures and be easily implemented in clinical settings. For instance, Czaplewske et al. fabricated a passive mechanical micro-sensor using a polysilicon micro-cantilever beam located along a microchannel. The beam deflection detected by an optical approach was induced by the liquid flow [16]. The sensitivity of such device can be improved by optimizing the microstructural shape and dimensions.

Herein, we report a passive micro-structured viscometer consisting of a suspending micro-membrane along a microchannel. The relatively large membrane area is configured for higher measurement sensitivity. Under a steady flow rate of the biosample, the membrane deflection can represent the liquid viscosity. The device is fabricated with biocompatible and optically transparent materials such that the membrane defection can be directly observed and measured under a microscope, which should be available in most clinical settings. Without any active flow-driving or sensing components, the low-cost device can be of one-time use, eliminating the concern of sample contamination. To further exhibit its applicability to biosamples, we applied the viscometer to measure blood samples with different volume ratios of the contained red blood cells.

## 2. Materials and Methods

### 2.1. Device Fabrication and Preparation

**Figure A1:**
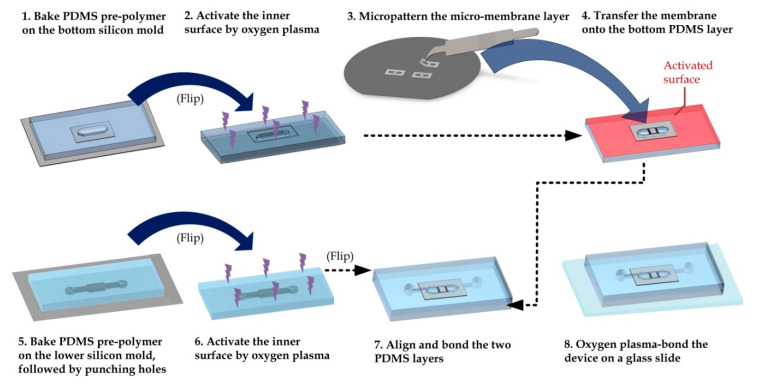
Fabrication process of the microfluidic viscometer.

The viscosity micro-sensor is fabricated based on photolithography and soft lithography [17,18]. This device contains three layers, with a suspending membrane sandwiched by an upper part and a lower part of a flow channel. The flow channel is fabricated by replica molding of polydimethylsiloxane (PDMS). A mold of the upper channel structure is fabricated with a 20-µm thick layer of negative photoresist (SU-8 2010, Microchem) patterned on a silicon wafer; whereas a mold of lower structure contains two micro-patterned layers of SU-8 photoresist (a 20-µm thick layer on top of a 25-µm thick layer) on a silicon wafer. The suspending membrane is fabricated by patterning a 25-µm thick SU-8 membrane on a silicon wafer, which is pre-silanized with vaporized trichloro (1H, 1H, 2H, 2H-perfluoro-octyl) silane (Sigma-Aldrich) to facilitate substrate release from them in the later process [19]. The resultant suspending micro-membrane is then peeled off from the silicon wafer with a razor blade and tweezers. To fabricate the device (Figure A1), PDMS pre-polymer (Sylgard-184, Dow Corning) is first prepared by mixing the monomer/curing agent with a 10:1 ratio. The pre-polymer is then poured onto both the upper and lower channel molds with a thickness of 5 mm and 2 mm, respectively, followed by baking both PDMS substrates at 80 °C overnight for the thorough crosslinking, which are then peeled off, cut along the device boundaries, and punched holes for the inlet and outlet. The lower channel substrate is placed on a flat surface with the channel side facing upward such that the micro-membrane can then be placed onto it with tweezers and aligned under a dissecting microscope. After air plasma treatment (energy: 10 kJ; Plasma Prep Ⅱ, SPI Supplies) for both half-channel sides, both substrates are aligned and bonded together under a dissecting microscope. The lower PDMS substrate of the assembled microchannel device is then bonded onto a glass slide (10127101P-G, Citoglas, Jiangsu, China) using air plasma.

### 2.2. Simulation

Finite element analysis was implemented using commercial software COMSOL Multiphysics (COMSOL 5.2a, Burlington, MA, USA) for obtaining shear force and dislocation of the suspending membrane under a steady flow of a liquid with different viscosity levels and driving flow rates. We considered water (density: 997 kg/m^3^; viscosity: 0.89 mPa·s) and mineral oil (density: 870 kg/m^3^; viscosity: 12 mPa·s) in the simulation. We adopted the laminar flow model instead of the whole Navier-Stokes equations solution for reducing the computational cost. Under a defined flow rate ranging from 0.05 to 0.5 mL/min for water and mineral oil, surrounding stresses around the suspending membrane were then computed by the simulation, in order to obtain the resultant shearing force on the membrane. Afterward, we set up another simulation for the deformation of the suspending membrane. We configured the material properties as the SU-8 photoresist (Young’s modulus: 2 GPa). We imposed the shear force at the membrane center to simulate the membrane deformation and displacement.

### 2.3. Image Capture and Processing

We applied an inverted microscope (TE300, Nikon) equipped with an sCMOS microscope camera (Zyla 4.2, Andor) for capturing phase-contrast images of the suspending membrane with a scale of (375 nm/pixel). An open source image processing software (ImageJ; NIH, Bethesda, MD, USA) was employed for further analyzing the microscopic images.

### 2.4. Blood Sample Preparation

We adopted bovine whole blood (adult bovine, 3.2% sodium citrate added, Hongquan Bio Inc, Guangzhou, China) in this work. We verified with a haemocytometer that the whole blood was composed of hemocytes with a volume ratio of ~40%. In addition, we prepared blood samples emulating the extracts from anemia and hematocytosis by regulating the haematocrit. In brief, we centrifuged the whole blood at 1400 rpm for 3 min to separate the plasma and hemocytes, with the upper half of the centrifuged blood extracted as the plasma and the remaining half as the blood with ~80% haematocrit. The plasma and the 80% haematocrit blood were then mixed with the unprocessed whole blood with an appropriate volume ratio to induce the blood samples with 20% (75% of plasma) and 60% haematocrit levels (25% of plasma), emulating the anemia and polycythemia conditions, respectively. It should be mentioned that cells in the prepared samples were sufficiently dispersed by a vortex mixer (Vortex mixers-SA8, Stuart Inc, Staffordshire, UK) before the experiments.

### 2.5. Statistics

All error bars in plots represent standard errors. P-values were obtained using Student’s t-test in Excel (Microsoft, Seattle, WA, USA). Asterisks represent a significant statistical difference (*p* < 0.05) between two groups of data in a plot.

## 3. Results and Discussion

### 3.1. Device Design

**Figure 1:**
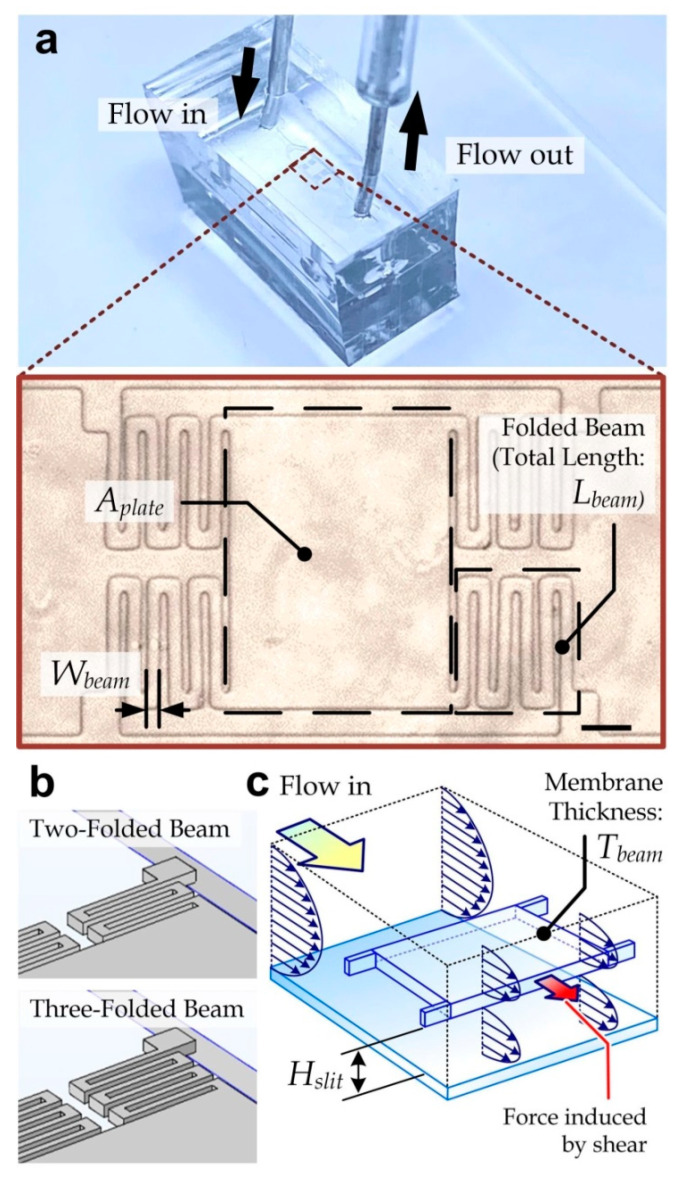
(**a**) Micro-viscometer containing a suspending micromembrane located along a microchannel. Inset: Enlarged microscopic image of the suspending membrane. Scale bar: 50 µm. (**b**) Designs of the two-folded beam (length: 800 µm) and three-folded beam (length: 1200 µm). (**c**) Key external forces acting on the suspending membrane. While the test sample is flowing along the channel, the shear stress over the membrane leads to the beam deflection and membrane displacement.

In this work, we developed a microfluidic viscometer containing a suspending micro-membrane as the sensing element for liquid viscosity. Briefly, the device contains a flow microchannel (height: 65 µm; width: 600 µm; length: 1500 µm) with one inlet and one outlet, and a suspending micro-membrane with a thickness of 25 µm in the middle of the channel, leaving 20 µm of spacing (*H_slit_*) above and below the membrane. The flow channel is fabricated by replica molding of two polydimethylsiloxane substrates for the upper and lower half of the microchannel. The suspending micro-membrane is generated by photolithography of a 25-µm thick layer of SU-8 epoxy. The shape of the membrane can be considered as a rectangular plate with a top-view area of *A_plate_* (500 µm × 375 µm) anchored to the microchannel by four folded cantilever beams, each with the same specific width *W_beam_* (=20 µm), thickness *T_beam_* (=25 µm), and total length *L_beam_*, as illustrated in Figure 1a. For the supporting beams we considered two designs with different total beam length: *L_beam_* = 800 µm for two-folded beams and *L_beam_* = 1200 µm for three-folded beams (Figure 1b). Notably, the design with four-folded beams was no longer considered because the beam stiffness is too low to support the membrane against deformation under its own weight.

During the viscosity measurement, we applied flow of the testing liquid along the micro-viscometer under a steady pressure from a compressed-air source. Meanwhile, the movement of the frontend of the liquid flow was detected by multiple optical proximity sensors placed along the outlet tubing, with each consisting of a light emitting diode, a single-pinhole mask, and a photoelectric sensor. The liquid flow rate can then be measured accordingly. Under the range of considered flow rates (≤0.5 mL/min), the flow was laminar and viscosity-dominant with a Reynolds number of ≪1. The force induced mainly by the viscous shear stress over the micro-membrane should lead to membrane displacement (Figure 1c), whose level is also balanced by the stiffness of the supporting folded beams. Apparently, the liquid viscosity can be obtained by measuring the membrane displacement.

The membrane displacement can be considered a function of the liquid viscosity and flow rate, as other parameters such as materials and dimensions are unchanged. Under a defined flow rate, the liquid viscosity can be converted from the membrane displacement, which is observed under a microscope in this work [20,21]. The liquid viscosity (*µ*) can be described as

(1) $$\mu \approx \frac{4K_{beam}H_{slit}{}^{3}}{A_{plate}}\times \frac{d}{Q}$$

where *d* is the membrane displacement; *Q* is the liquid flow rate, *H_slit_* is the spacing above or below the membrane; *A_plate_* is the top-view membrane area; and *K* is the”spring constant” of each folded beam, which can be roughly approximated by

(2) $$K_{beam}\approx \frac{4ET_{beam}{}^{3}W_{beam}}{L_{beam}{}^{3}}$$

where *E* is Young’s modulus of SU-8 epoxy; *L_beam_*, *W_beam_*, and *T_beam_* are the total length, width, and thickness of one folded micro-beam, respectively.

### 3.2. Simulation Analysis

**Figure 2:**
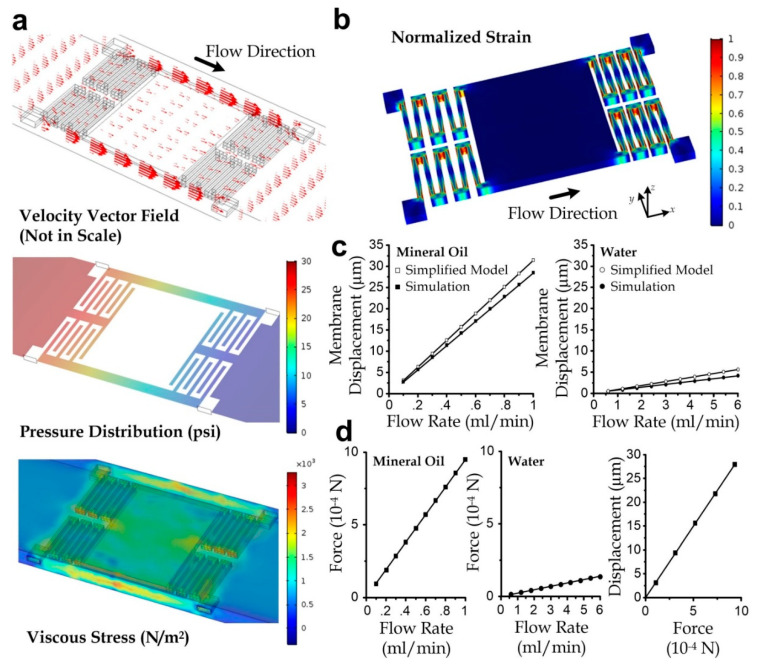
(**a**) Velocity, pressure, and viscous stress profiles around the micromembrane supported by the three-folded beams at a flow rate of 0.3 mL/min. (**b**) Strain distribution of the suspending membrane along the microchannel. (**c**) Membrane displacements as a function of the flow rate, calculated by simulation and the simplified model. (**d**) Resultant force acting on the membrane as functions of the flow rate and membrane displacement.

We performed a simulation to verify the relations described by Equations (1) and (2). Two geometry designs for a flow channel containing either the two-folded beams or three-folded beams were considered in this task. For each design, we configured the flowing liquid to be water (viscosity: 0.89 mPa·s) and mineral oil (viscosity: 12 mPa·s). We simulated for the steady stress distribution over the micro-membrane surface under a defined flow rate of either water or mineral oil, ranging from 0.05 mL/min to 0.5 mL/min. For instance, the velocity, pressure, and viscous stress profile for the three-folded beam device design under a mineral oil flow rate of 0.3 mL/min is illustrated in Figure 2a.

Furthermore, we computed the membrane deformation by considering the suspending layer, which is the micromembrane anchored by either the two-folded or the three-folded beams. In each case of defined membrane design, flowing liquid, and flow rate, the stress distribution obtained from the previous flow simulation was then mapped as boundary conditions in this task. The material properties of the SU-8 photoresist were set as hyperelastic, with the key parameters adopted from the accompanying material database in the software. As a demonstration, the strain profile of a 3-folded membrane corresponding to the 0.3 mL/min flow of mineral oil is illustrated in Figure 2b. The membrane displacement as a function of flow rate is plotted in Figure 2c. For comparison, it also includes the analytical results calculated using Equation (2) and *F* = *Kd*, where the resultant force *F* is the area integral of stress over the membrane surface. A reasonable agreement between the simulation and the analytical approximation in terms of the gradients in the plot can be observed; the mismatch is possibly due to the approximation of the spring constant and inaccuracy of parameters such as dimensions and material properties. A correction factor of 1.091 can be applied to scale the analytical approximations to the simulated membrane displacements with an accuracy of >99.8%, which was obtained from the ratio of the average experimental value to the simulated value. In addition, owing to the very low Reynolds number flow, the resultant force (*F*) as an area integral of stress had a linear relationship with the applied flow rate (*Q*) and membrane displacement (*d*), as shown in Figure 2d, agreeing with Equation (1).

### 3.3. Sensor Characterization

**Figure 3:**
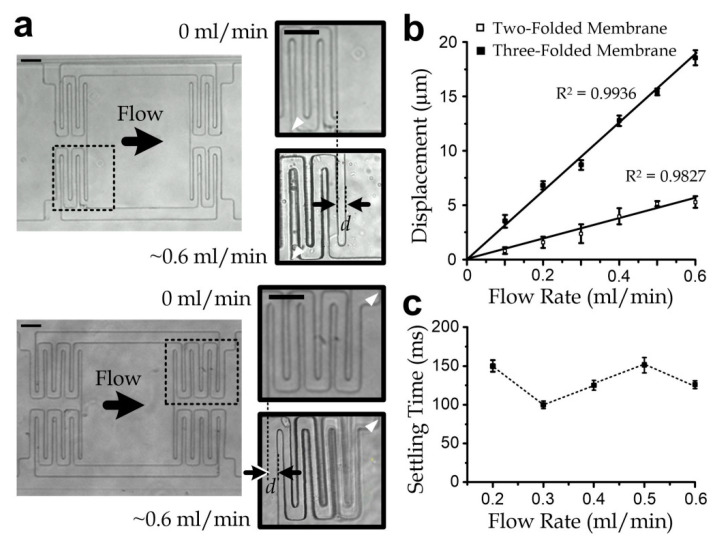
(**a**) Microscopic images of the two-folded and three-folded membranes. Inset: magnified microscopic images of the folded beam regions highlighted by the dotted boxes for the cases of no flow and flowing mineral oil at a flow rate of ~0.6 mL/min. The membrane displacements are denoted as ‘*d*’. White arrows indicate the anchor positions. All scale bars: 50 µm. (**b**) Comparison of the membrane displacements of the two-folded and three-folded designs under different flow rates of mineral oil. (**c**) Settling time of the membrane displacement under flow rates of mineral oil up to 0.6 mL/min. All error bars in this figure are standard errors (*N* ≥ 5).

**Figure A2:**
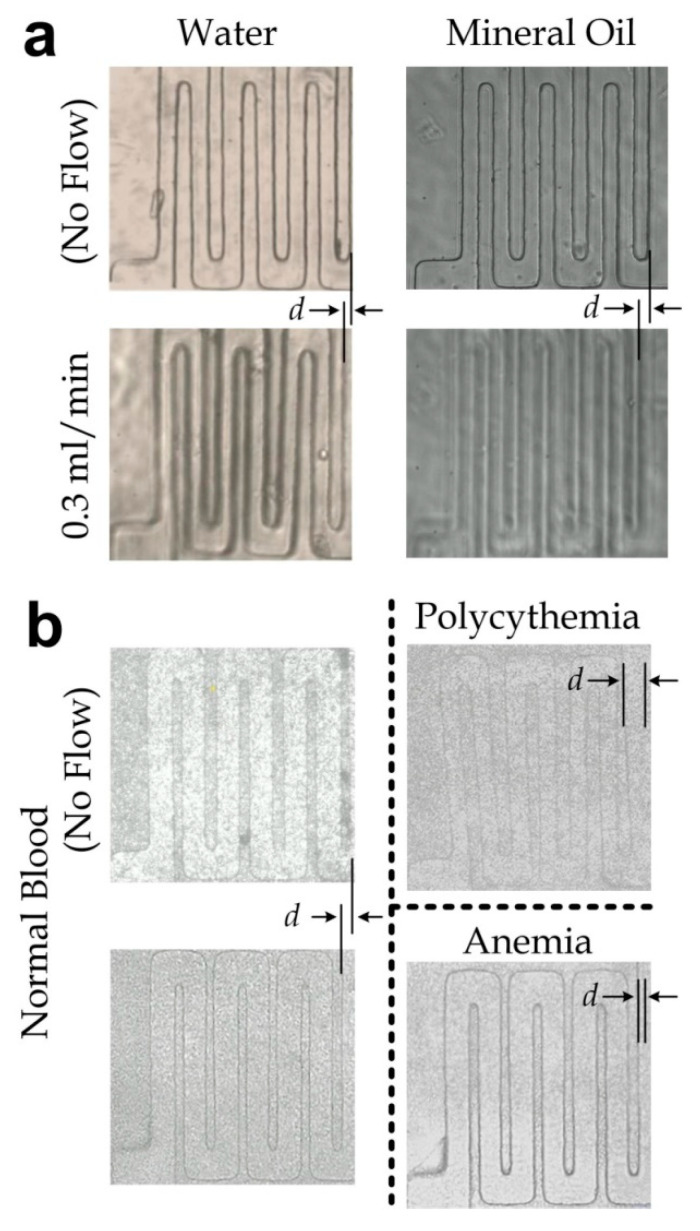
(**a**) Microscopic images of membrane displacements without and with the flow of water and mineral oil. The flow rate was chosen as 0.3 mL/min for both liquids for comparison. (**b**) Membrane displacements under flows (0.3 mL/min) of blood samples with different hematocrit conditions (anemia, normal, and polycythemia).

We investigated the measurements of the two-folded and three-folded beam designs. We selected light mineral oil (M5310, Sigma-Aldrich, St. Louis, MO, USA) as the testing liquid. The membrane displacement under a flow rate ranging 0–0.6 mL/min of either design was imaged under an optical inverted microscope (TE300, Nikon, Melville, NY, USA), as illustrated in Figure 3a and Figure A2a. The driving pressure was first adjusted to obtain the required flow rate in each measurement for this task. All the measured membrane displacements, which were quantified with the captured microscopic images, are summarized in Figure 3b. The high linearity (R^2^ > 0.98) between the membrane displacement and the flow rate reflects the high measurement consistency. According to the slopes of the fitting lines, the liquid viscosity can be obtained by Equations (1) and (2) with a scaling factor of 1.091, as explained above. The viscosity of mineral oil measured by the two-folded and three-folded beam designs was 1.259 × 10^−2^ ± SE 9.88 × 10^−5^ Pa·s and 1.098 × 10^−2^ ± SE 1.2 × 10^−4^ Pa·s, respectively, agreeing well with the value (0.012 Pa·s) provided in the product specifications. Generally, smaller beam stiffness (i.e., three-folded beam design) should be chosen for larger membrane deflection and the corresponding sensitivity because of the longer beam length according to Equation (2). The width and thickness of the folded beam were set to be small for the higher sensitivity but not too small for supporting the freestanding micromembrane. Furthermore, the membrane deformation stabilized in 200 ms in all the measurements (Figure 3c), suggesting that a continuous flow after 1 s can induce a promising measurement result. Recalling the liquid front detection scheme along the outlet tubing, the flow rate can be measured after such 1 s of continuous flow. This implies that a sample volume of 100 µL of mineral oil is more than sufficient for the measurement. In the practical implementation, a higher flow rate can be adopted for higher measurement precision as long as the steady flow duration is at least 1 s.

**Figure 4:**
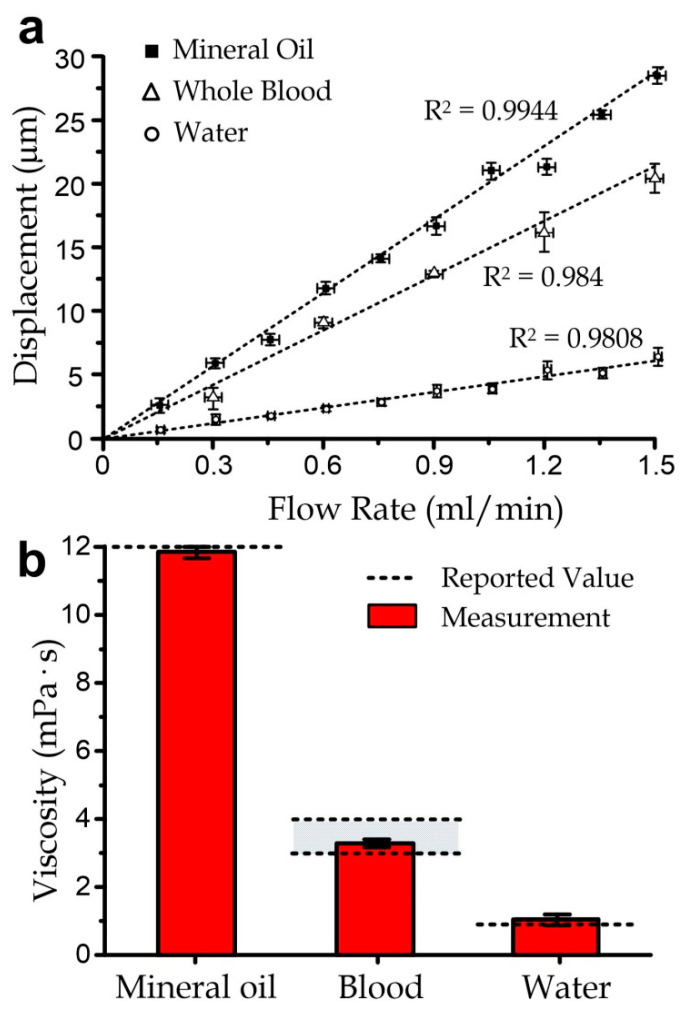
(**a**) Membrane displacement of the microfluidic viscometer as a function of the flow rate under flows of mineral oil, water, and bovine whole blood. (**b**) Resolved viscosity of the measured liquids compared with the reported values from the previous works. All error bars are standard errors of repeated measurements (*N* ≥ 5).

We applied the three-folded beam design to measure also the viscosity levels of distilled water and whole bovine blood (adult bovine with 3.2% sodium citrate) under different flow rates, as summarized in Figure 4. Distilled water was measured at room temperature, and the blood was preheated at 36.5 °C in water right before measurement. The flow rate was set as 0–6 mL/min for distilled water and 0–1.5 mL/min for whole blood. The measured water viscosity was 9.6 × 10^−4^ ± SE 3.6 × 10^−5^ Pa·s, with a good agreement (~99.48% accuracy) with the reported value of 9.55 ×10^−4^ Pa·s, measured in the laboratory at 22 °C. Besides, the measured blood viscosity was 3.08 × 10^−3^ ± SE 3.85 × 10^−4^ Pa·s, which had a reasonable agreement (~97.3% accuracy) with the reported range of 3 × 10^−3^–4 × 10^−2^ Pa·s [22].

### 3.4. Measurement of Blood Samples with Varied Viscosity

To further demonstrate bio-related applications, we measured a few groups of pre-mixed blood samples (volume: 50 µL) with different haematocrit levels, which are the volume percentages of red blood cells in blood. A flow rate of ≥0.3 mL/min applied to blood in this work induced a shear rate of >6250 s^−1^. Notably, although blood is a non-Newtonian fluid, it shows Newtonian fluid behavior at high shear rates (>500 s^−1^) [22,23]. The haematocrit is an important health indicator, as it largely determines the oxygen-carrying capacity of blood. Anemia and polycythemia describe blood containing significantly more of fewer red blood cells, respectively. The blood viscosity is well known to correlate with the haematocrit, as also demonstrated in Figure A2b. For instance, normal human or bovine blood has ~40% haematocrit and a viscosity of 3–4 × 10^−3^ Pa·s, as mentioned previously. A hematocrit of 60% (polycythemia) induces a viscosity of 6–8 × 10^−3^ Pa·s; whereas a haematocrit of 20% (anemia) induces a viscosity of ~2 × 10^−3^ Pa·s [24]. 

**Figure 5:**
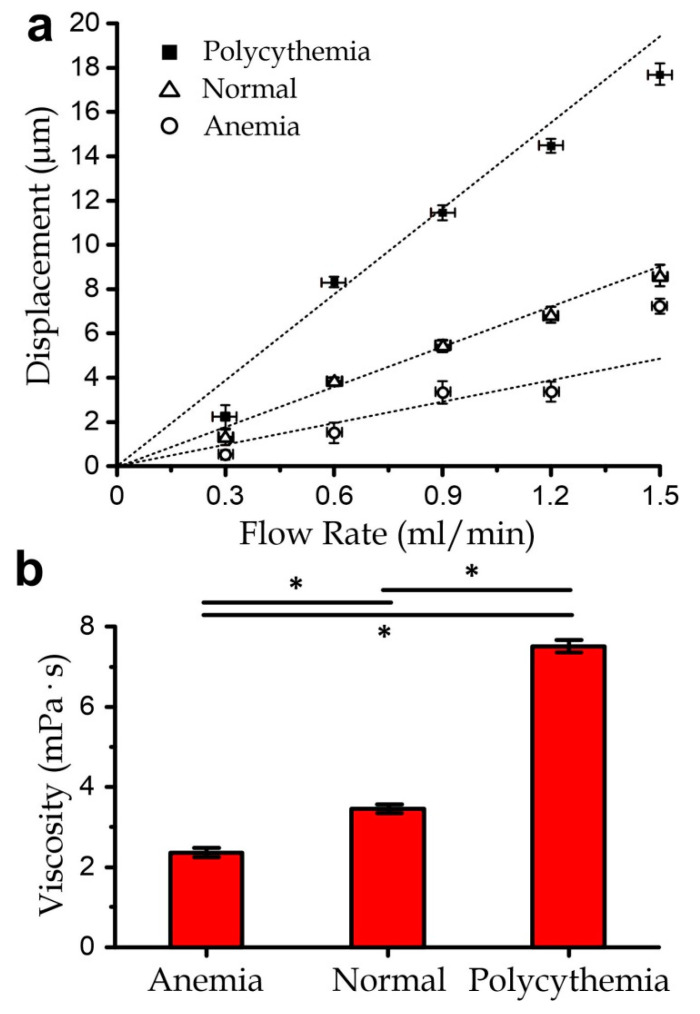
(**a**) Micromembrane displacements for anemia, normal, and polycythemia bovine blood samples for different sample flow rates. Dotted lines are fitting lines. All error bars are standard errors (*N* ≥ 5). (**b**) Comparison of measured viscosity values for different blood samples. All error bars are standard errors. Asterisks indicate a *p*-value < 0.05.

In the experiments, we prepared blood samples with a modulated haematocrit level of 20% or 60% as described in the Methods section and kept the samples at 37 °C in a water bath; the measurement results for the blood samples with a volume of 50 µL each are plotted in Figure 5. Our results indicate good agreements with the reported values that 20% haematocrit induces a blood viscosity of 2.74 × 10^−3^ ± SE 2.7 × 10^−4^ Pa·s, and that 60% haematocrit induces a blood viscosity of 7.56 × 10^−3^ ± SE 3.7 × 10^−4^ Pa·s. This implies that the micro-membrane viscometer can be applied to measure patients’ blood with a volume of ~50 µL and estimate the haematocrit level. It should be worth mentioning that each of the 50-µL bio-samples can be recollected and used in other downstream bioassays and genetic/biochemical processing. For instance, the recollected blood can be further quantified for cytokine concentrations; and each cytokine type requires only a volume of <5 µL [25,26]. The monocytes in blood can be isolated for further analyses [27,28]. The red blood cells can also be recycled for other biophysical measurements [29,30]. On the other hand, for the practical implementation, the microfluidic viscometer should be placed on a stable workbench without any vibrating medical equipment (e.g., pumps, printers) to avoid any unnecessary disturbance to the micromembrane deflection.

## 4. Conclusions

We have developed a micromembrane-based viscometer for the measurements of bio-fluids (e.g., blood) with simple operation procedures. Such displacement is proportional to the sample viscosity and can be easily observed under a regular bright-field microscope. The required sample volume (<50 µL) is much smaller than required in traditional approaches such as the rotational and capillary methods; hence, the reported micro-viscometer can be applied to various clinical bio-samples, which often can only be extracted with a limited volume. We configured the operation parameters and the shape and dimensions of the micromembrane through analytical study, simulation, and experimental verification. We determined a correction factor to match a simplified but intuitive analytical prediction of the membrane displacement to the simulation results; this corrected model had an accuracy of >98% for the viscosity measurement of water and mineral oil. The membrane displacement under a steady driving pressure can stabilize within 200 ms for reliable measurement. Besides, the micro-viscometer was fabricated with low-cost and biocompatible polymeric materials; hence, it can be considered for one-time use without causing any biosample contamination. In essence, we have demonstrated that the viscometer can measure blood samples with different haematocrit levels. Considering also that the micromembrane-based measurement does not induce any noticeable effects on the biosamples, these can be recollected for other assays after the viscosity measurement. Overall, the reported microfluidic viscometer is highly compatible with biomedical applications including bioassays, disease monitoring, and diagnosis.
